# Supplementary material for: Prediction of risk scores for colorectal cancer patients from the concentration of proteins involved in mitochondrial apoptotic pathway
Source: PLoS One. 2019 Sep 9;14(9):e0217527. doi: 10.1371/journal.pone.0217527 (PMC6733437; doi:10.1371/journal.pone.0217527)
Supplement: S1 File — Fig A, Stratification of high and low risk CRC patients based on OS using β1 (i) Patients with β1 < mean(β1) are classified as High Risk as compared to patients with β1 ≥ mean(β1). (ii) Patients with β1 < median(β1) are classified as High Risk as compared to patients with β1 ≥ median(β1). Fig B, Stratification of high and low risk CRC patients based on OS using β2 (i) Patients with β2 < mean(β2) are classified as High Risk as compared to patients with β2 ≥ mean(β2). (ii) Patients with β2 < median(β2) are classified as High Risk as compared to patients with β2 ≥ median(β2). Fig C, Stratification of high and low risk CRC patients based on OS using β3(i) Patients with β3 < mean(β3)re classified as High Risk as compared to patients with β3 ≥ mean(β3). (ii) Patients with β3 < median(β3) are classified as High Risk as compared to patients with β3 ≥ median(β3). Fig D, Stratification of high and low risk CRC patients based on OS using β4(i) Patients with β4 < mean(β4)re classified as High Risk as compared to patients with β4 ≥ mean(β4). (ii) Patients with β4 < median(β4) are classified as High Risk as compared to patients with β4 ≥ median(β4). Fig E, Stratification of high and low risk CRC patients based on OS using β5(i) Patients with β5 < mean(β5)re classified as High Risk as compared to patients with β5 ≥ mean(β5). (ii) Patients with β5 < median(β5) are classified as High Risk as compared to patients with β5 ≥ median(β5). Fig F, Multivariate COX-PH hazard analysis for risk estimation in the CRC patients based on β1 (i) At mean cutoff. and (ii) At median cutoff. Here right tumor location—hepatic flexure, caecum, ascending, traverse; left tumor location—splenic flexure, descending, sigmoid and rectal—rectum, rectosigmoid. Fig G, Multivariate COX-PH hazard analysis for risk estimation in the CRC patients based on β2 (i) At mean cutoff. and (ii) At median cutoff. Here right tumor location—hepatic flexure, caecum, ascending, traverse; left tumor location—splenic flexure, desc [file pone.0217527.s001.docx]

**S1: Supplementary file**

**Prediction of risk scores for colorectal cancer patients from**

**the concentration of proteins involved in mitochondrial**

**apoptotic pathway.**

Anjali Lathwal^1,‡^, Chakit Arora^1,‡^, Gajendra P. S. Raghava^1*^

1Department of Computational Biology, Indraprastha Institute of Information

Technology, New Delhi, India

‡These authors contributed equally to this work.

* raghava@iiitd.ac.in


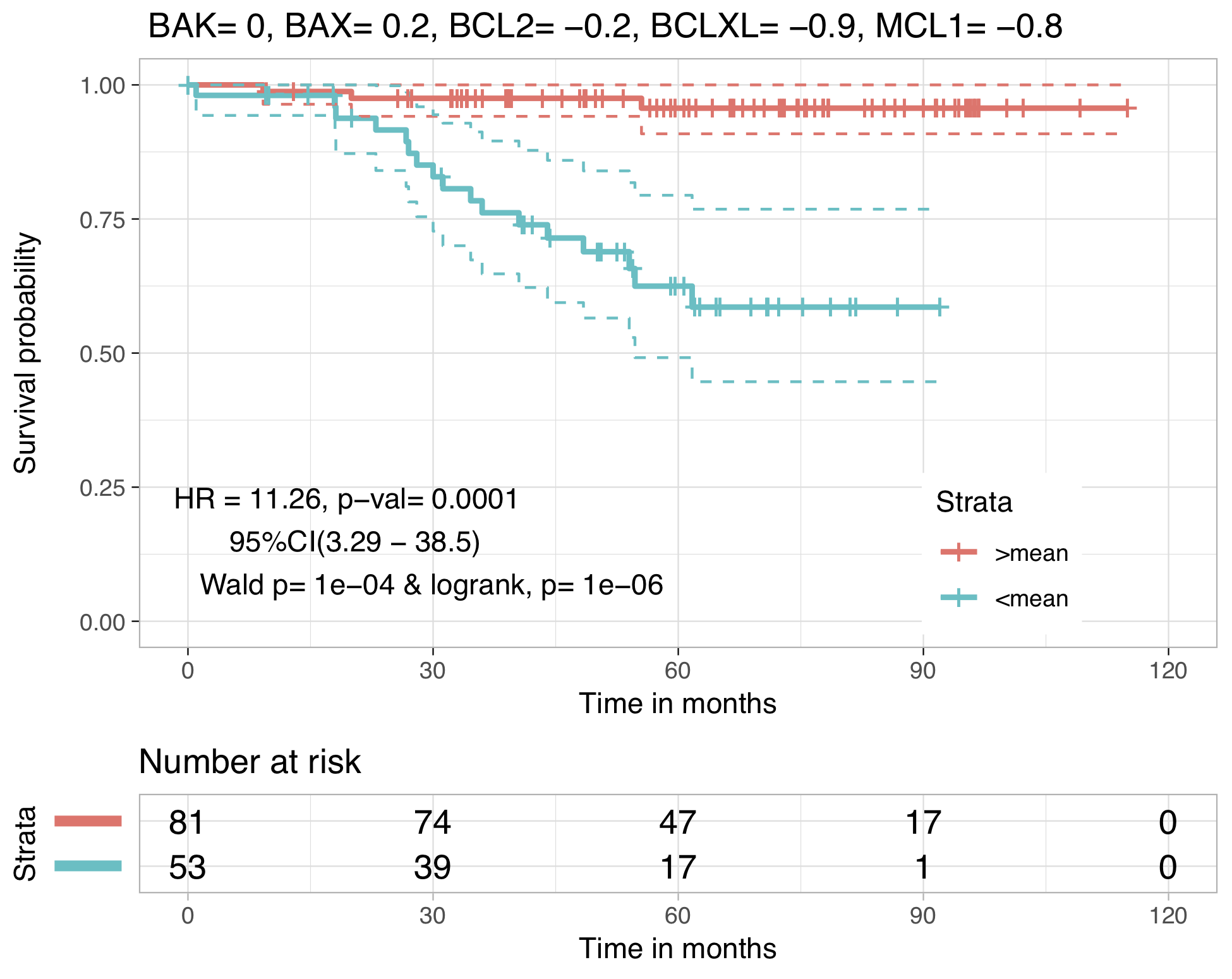

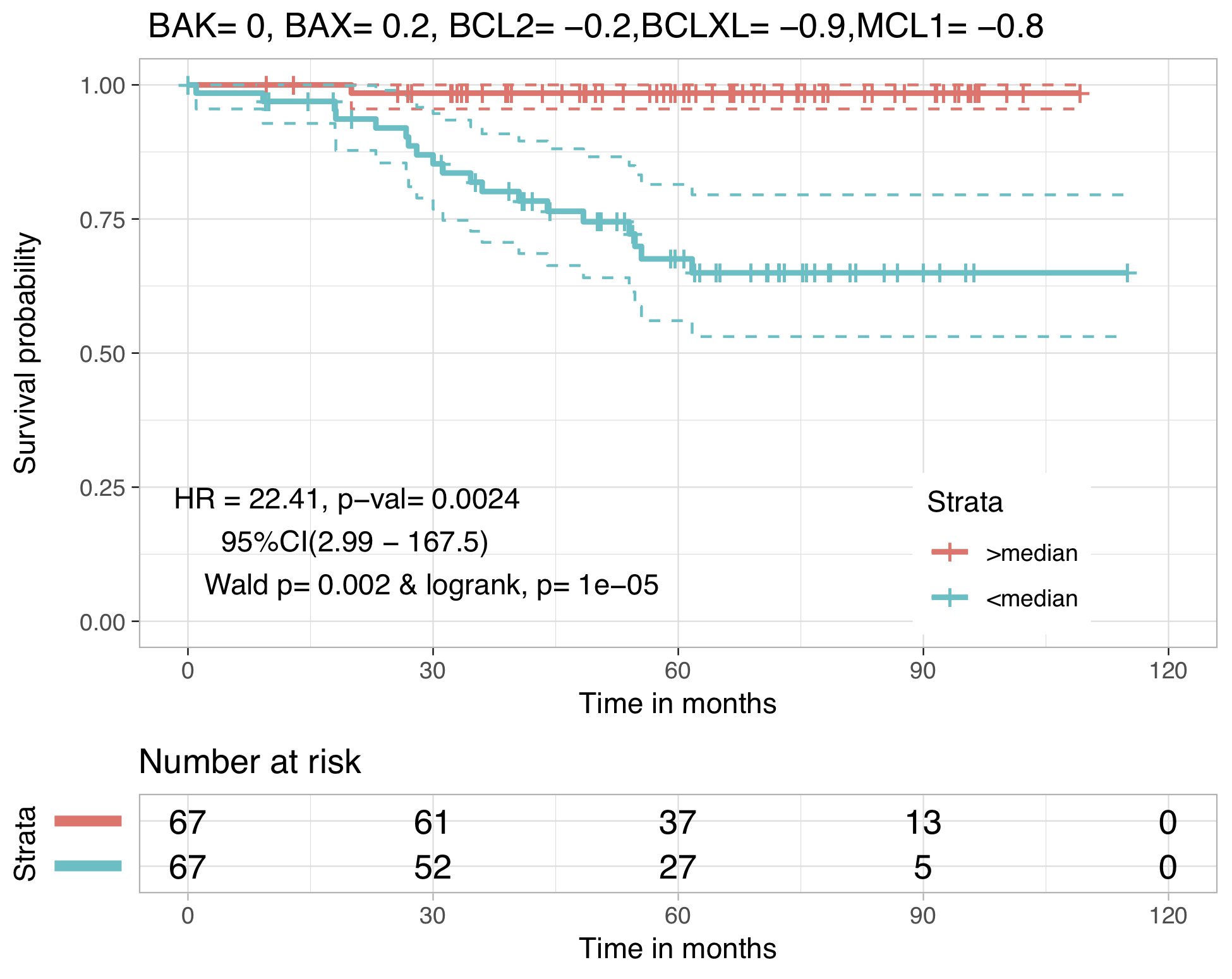


(i) (ii)

**S1 Fig A**


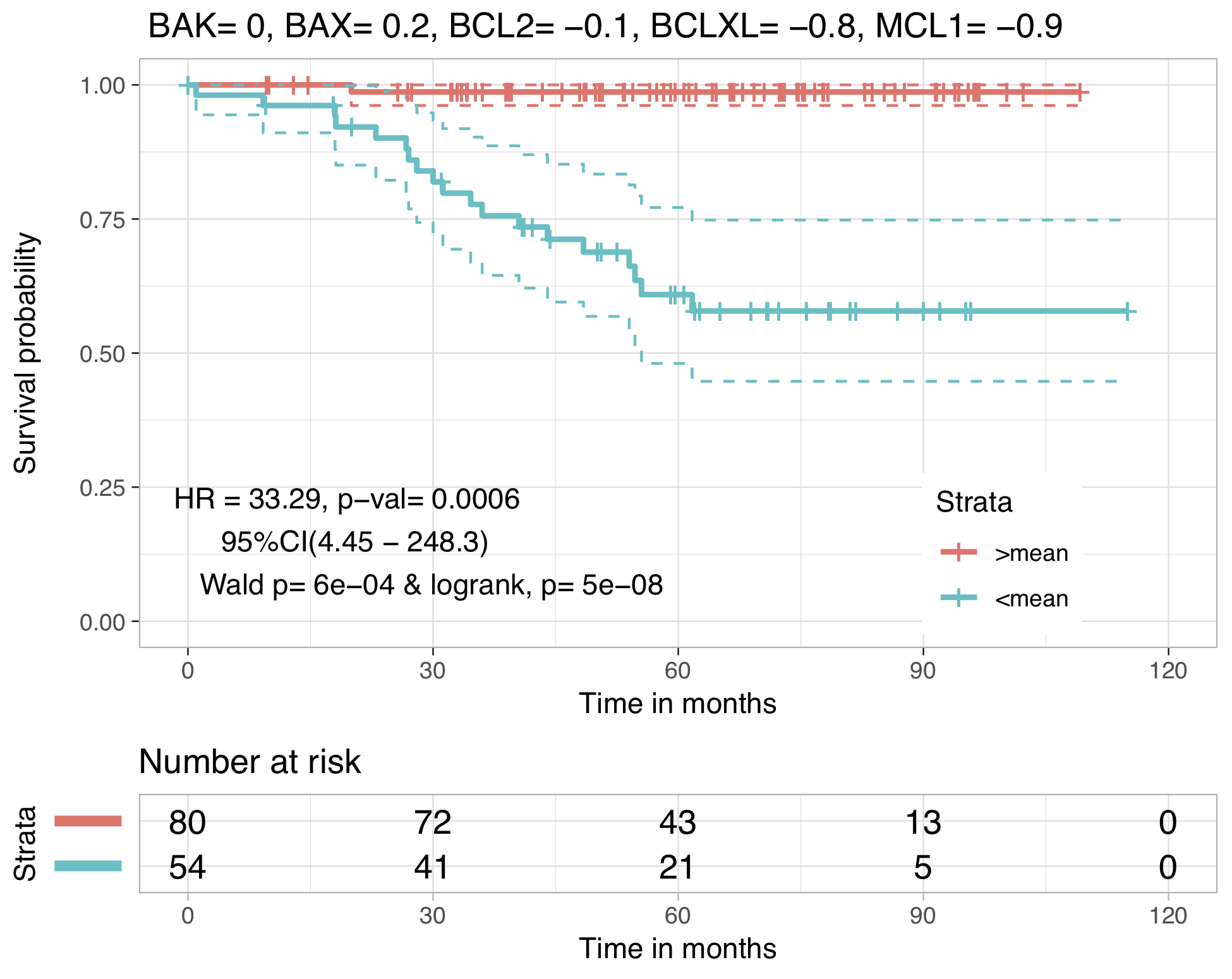

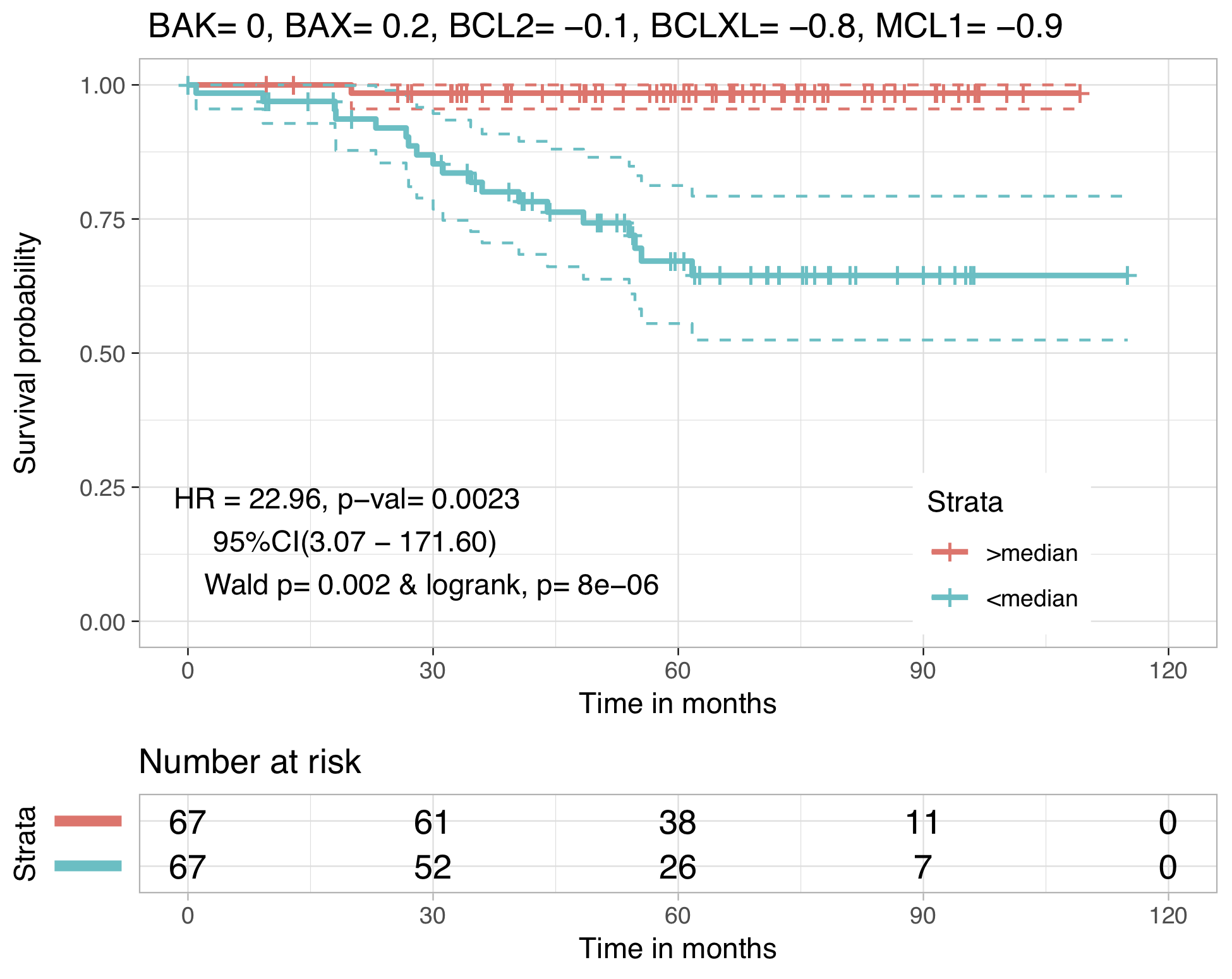


(i) (ii)

**S1 Fig B**


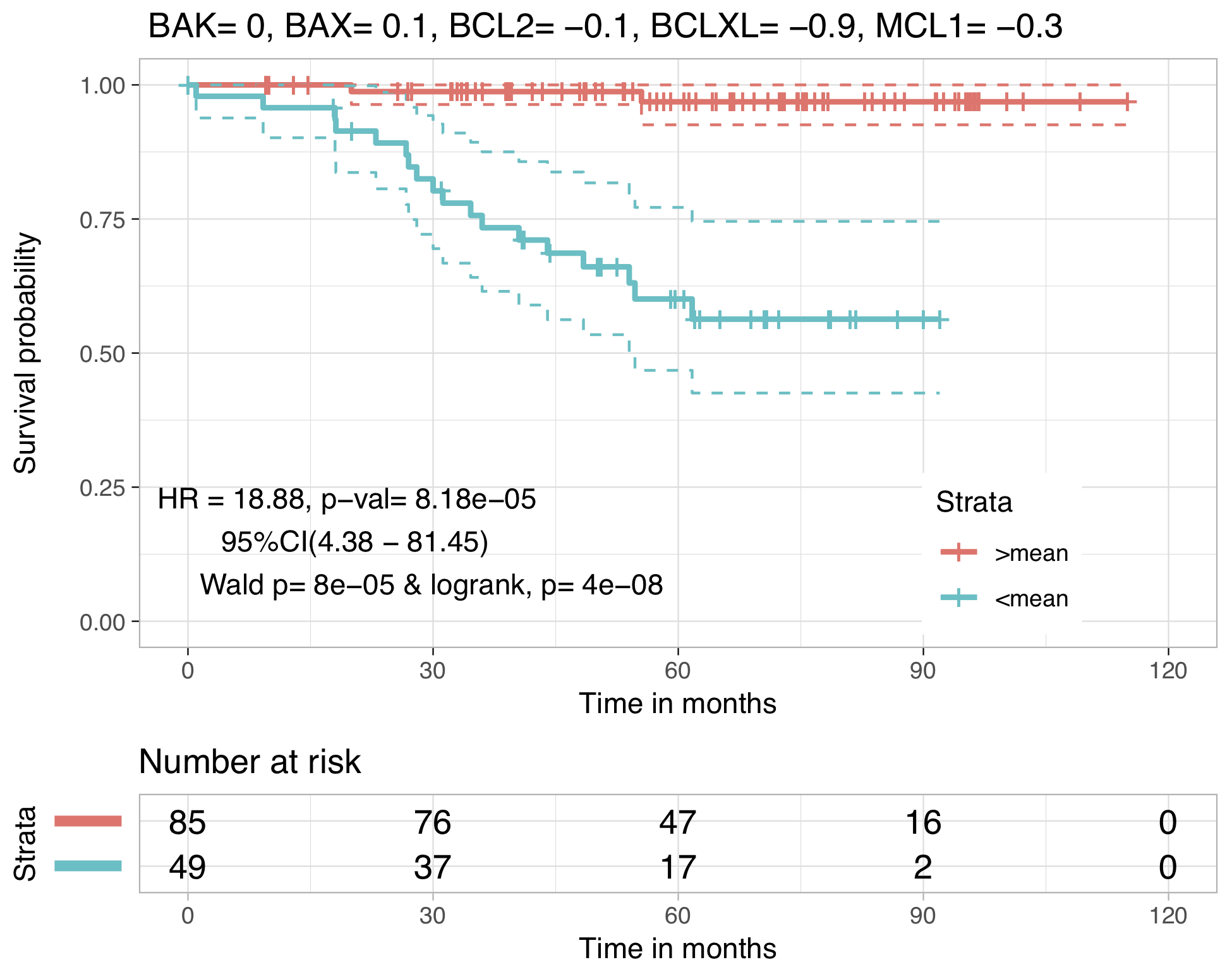

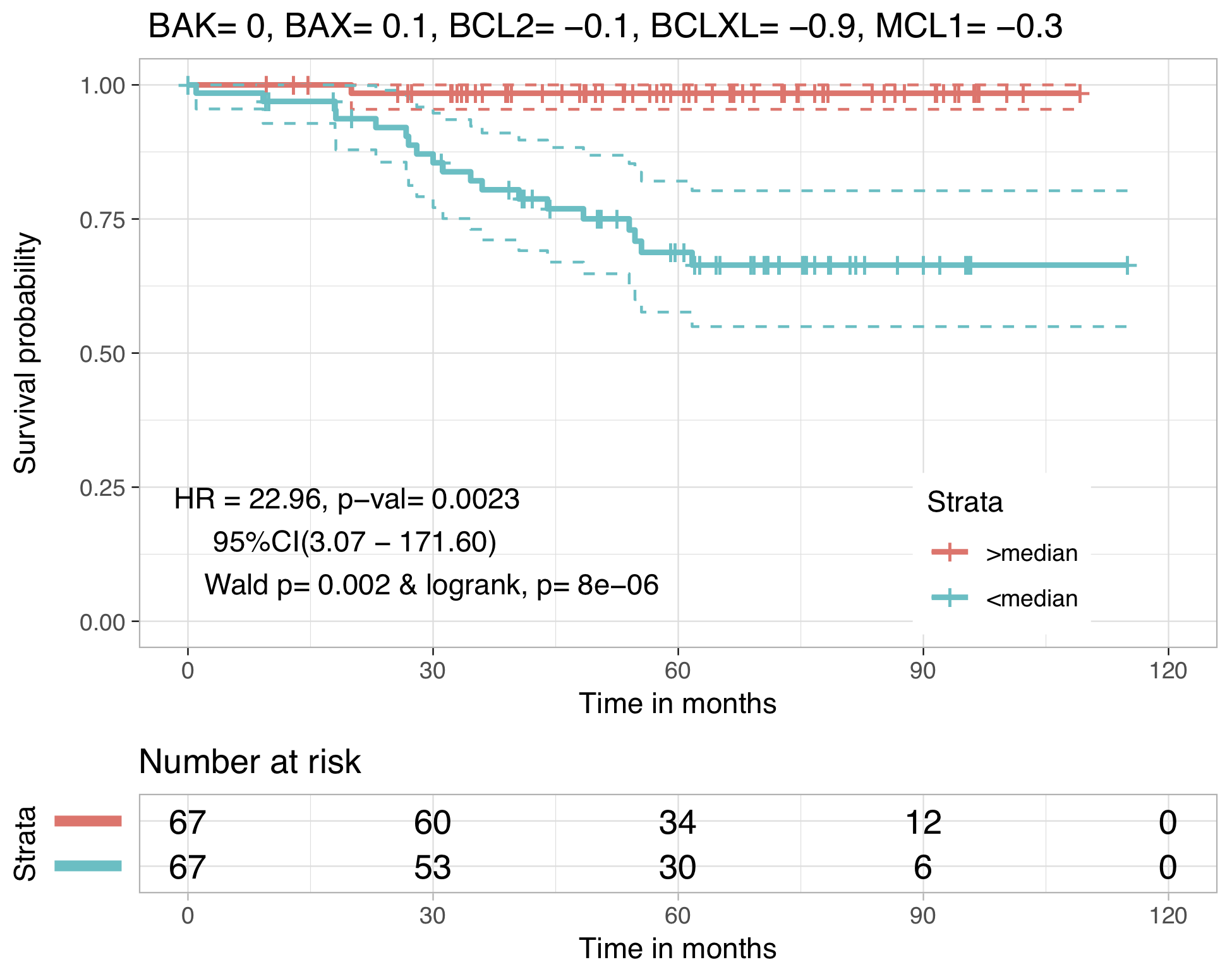


(i) (ii)

**S1 Fig C**


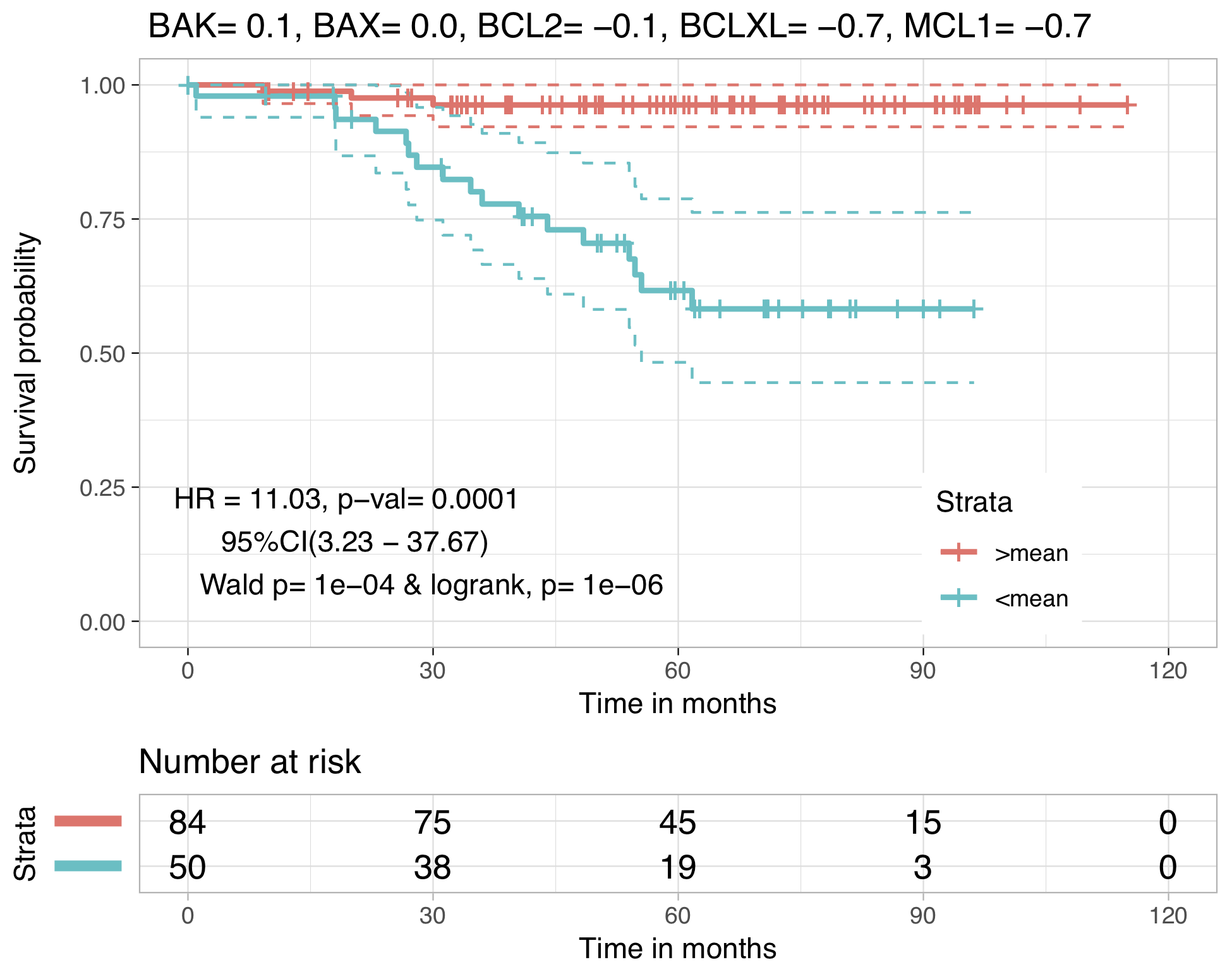

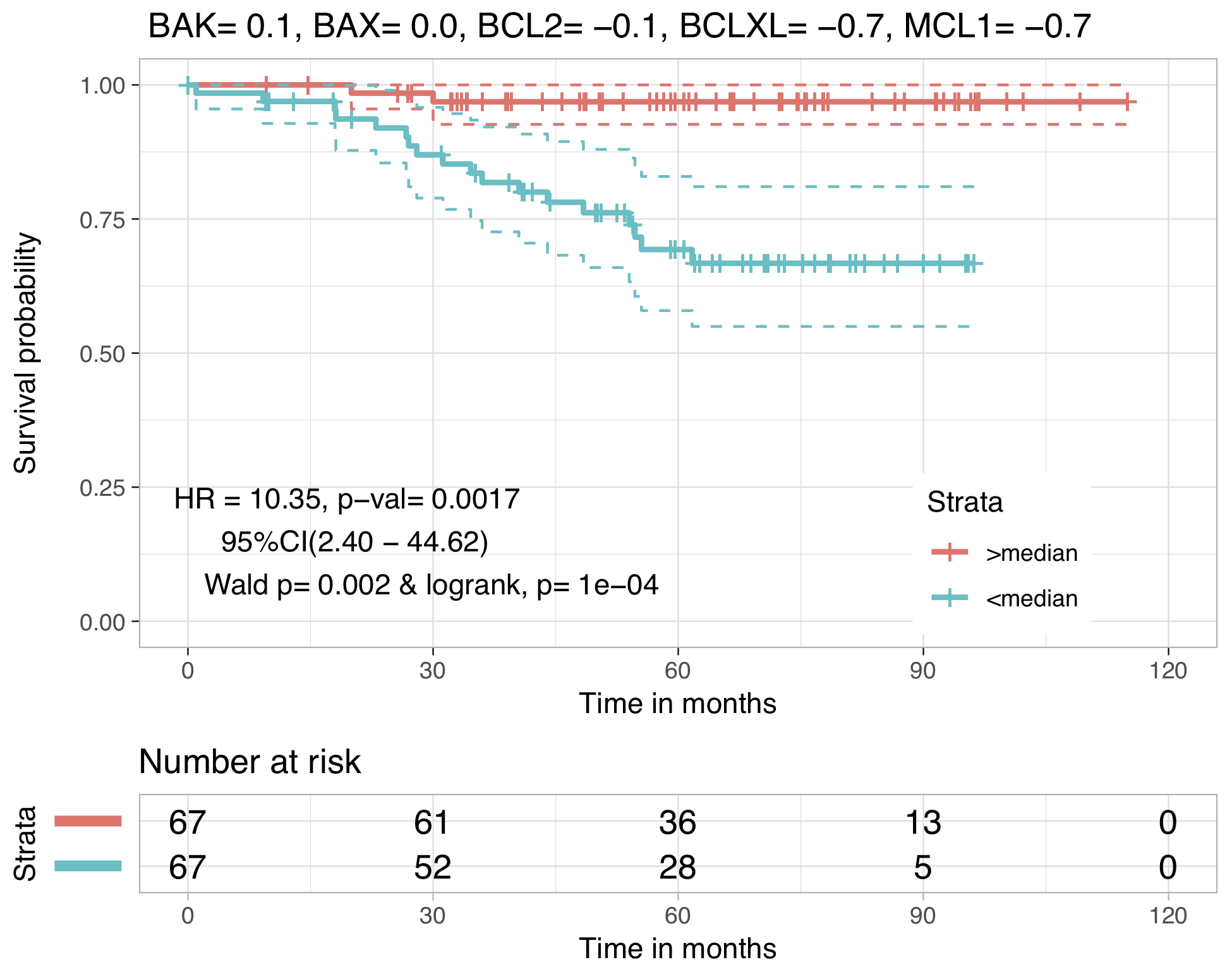


(i) (ii)

**S1 Fig D**


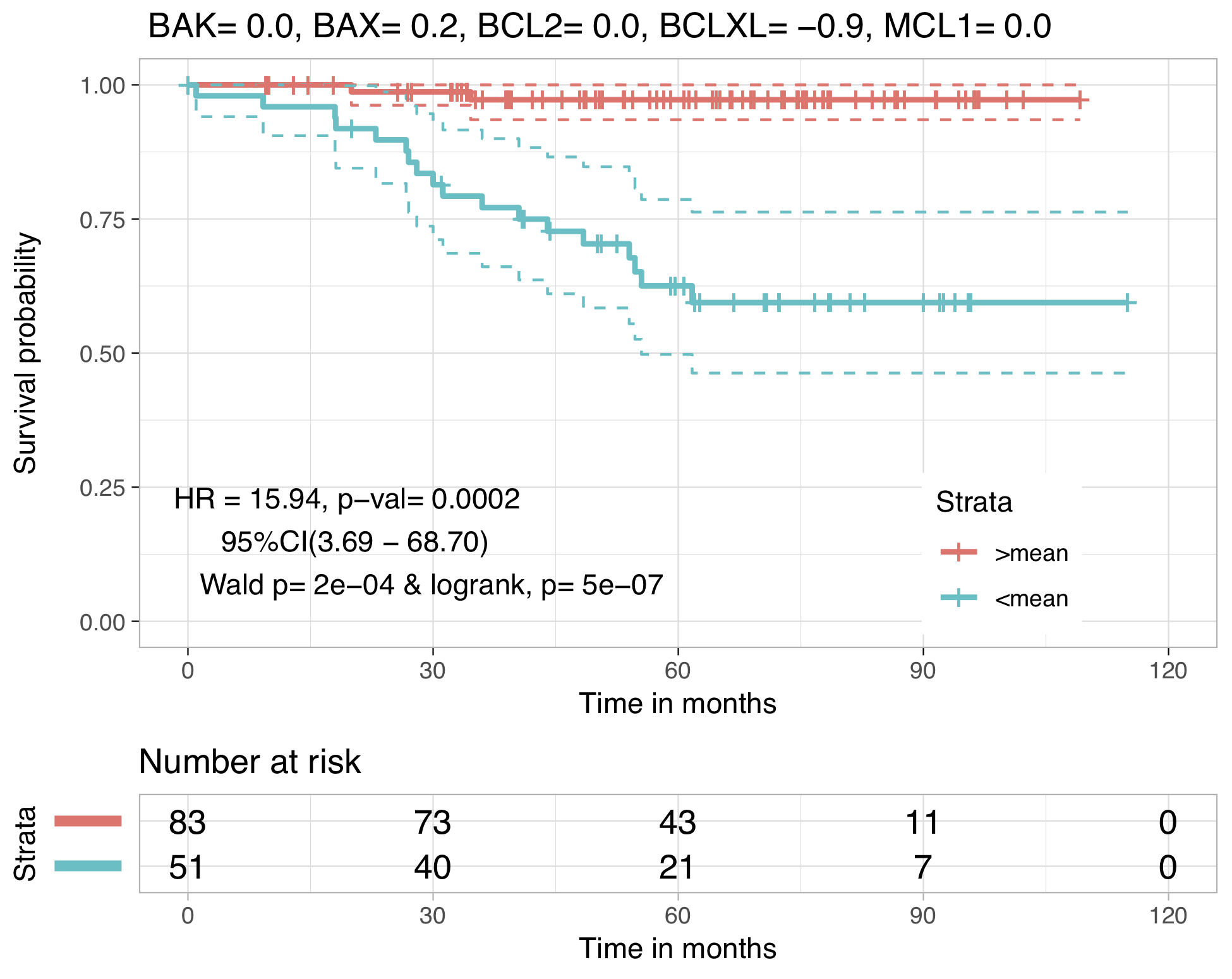

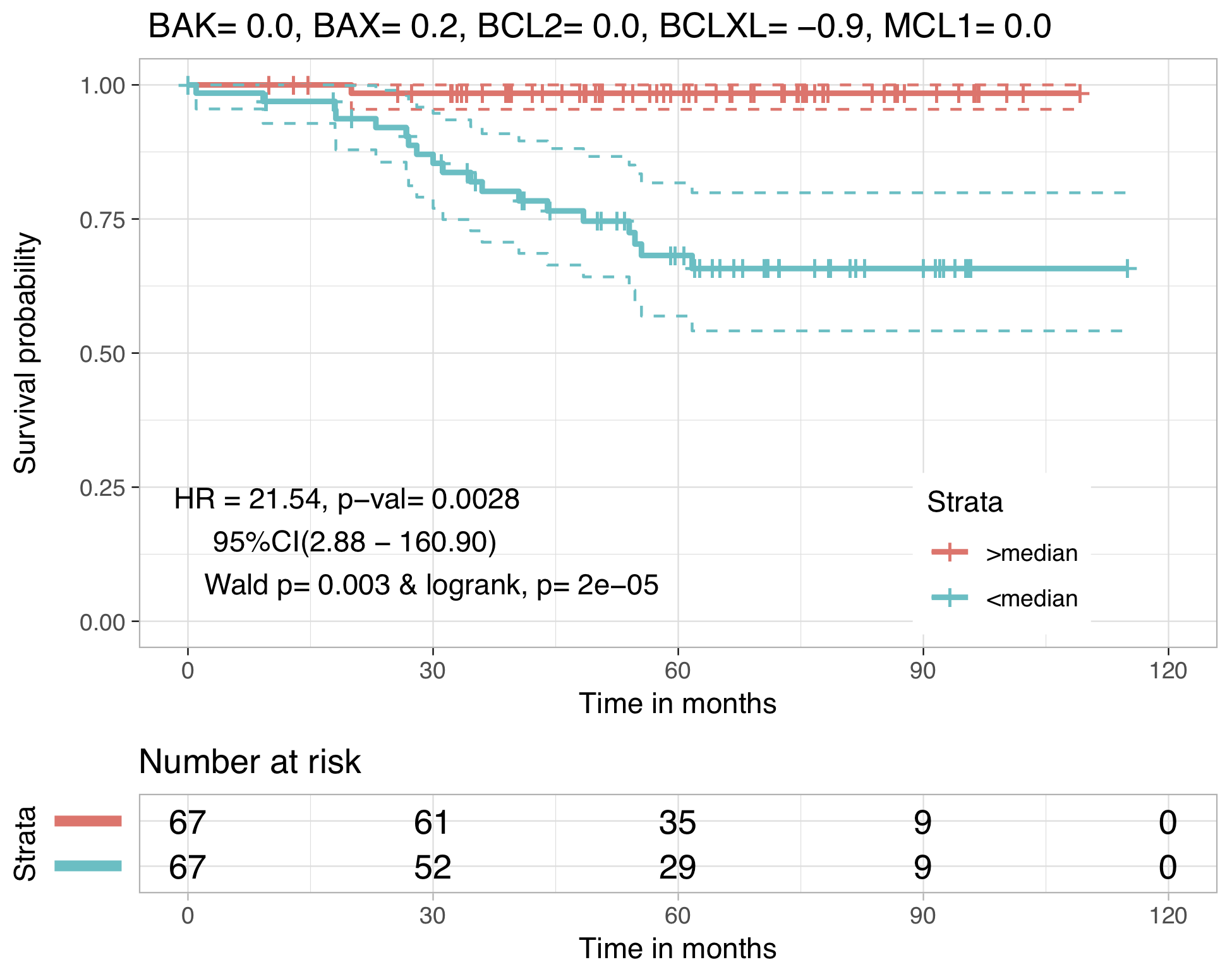


(i) (ii)

**S1 Fig E**


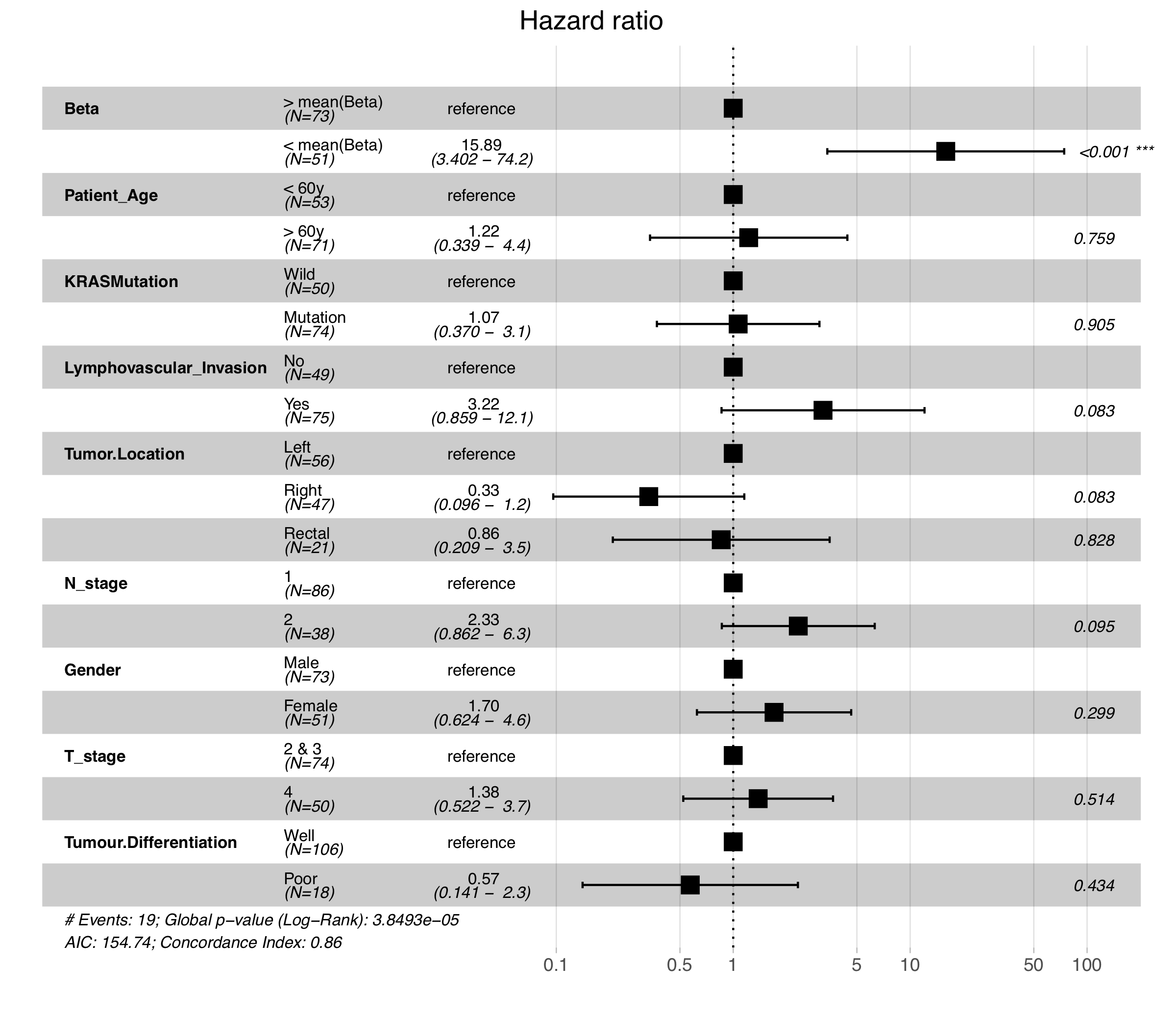

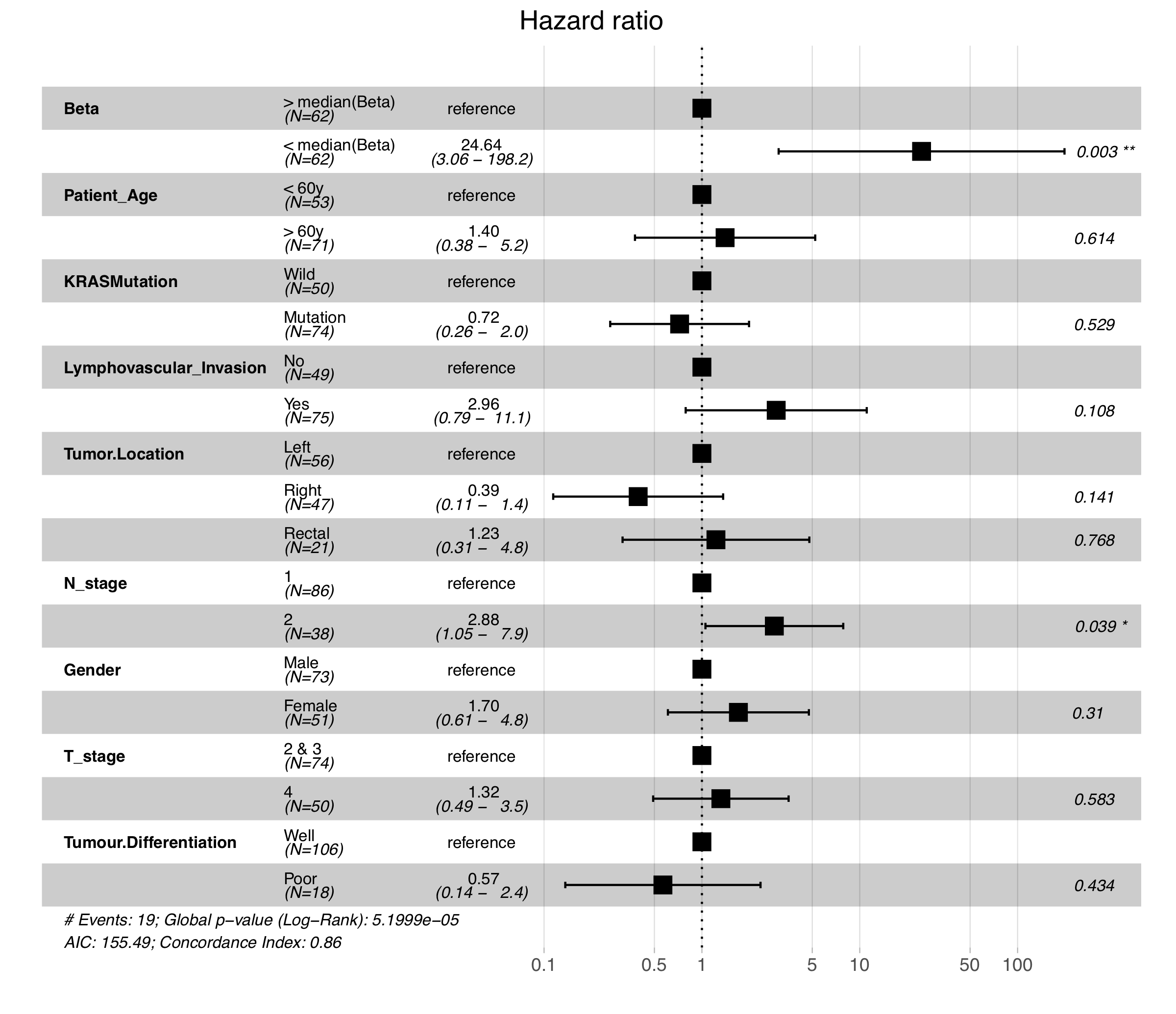


(i) (ii)

**S1 Fig F**


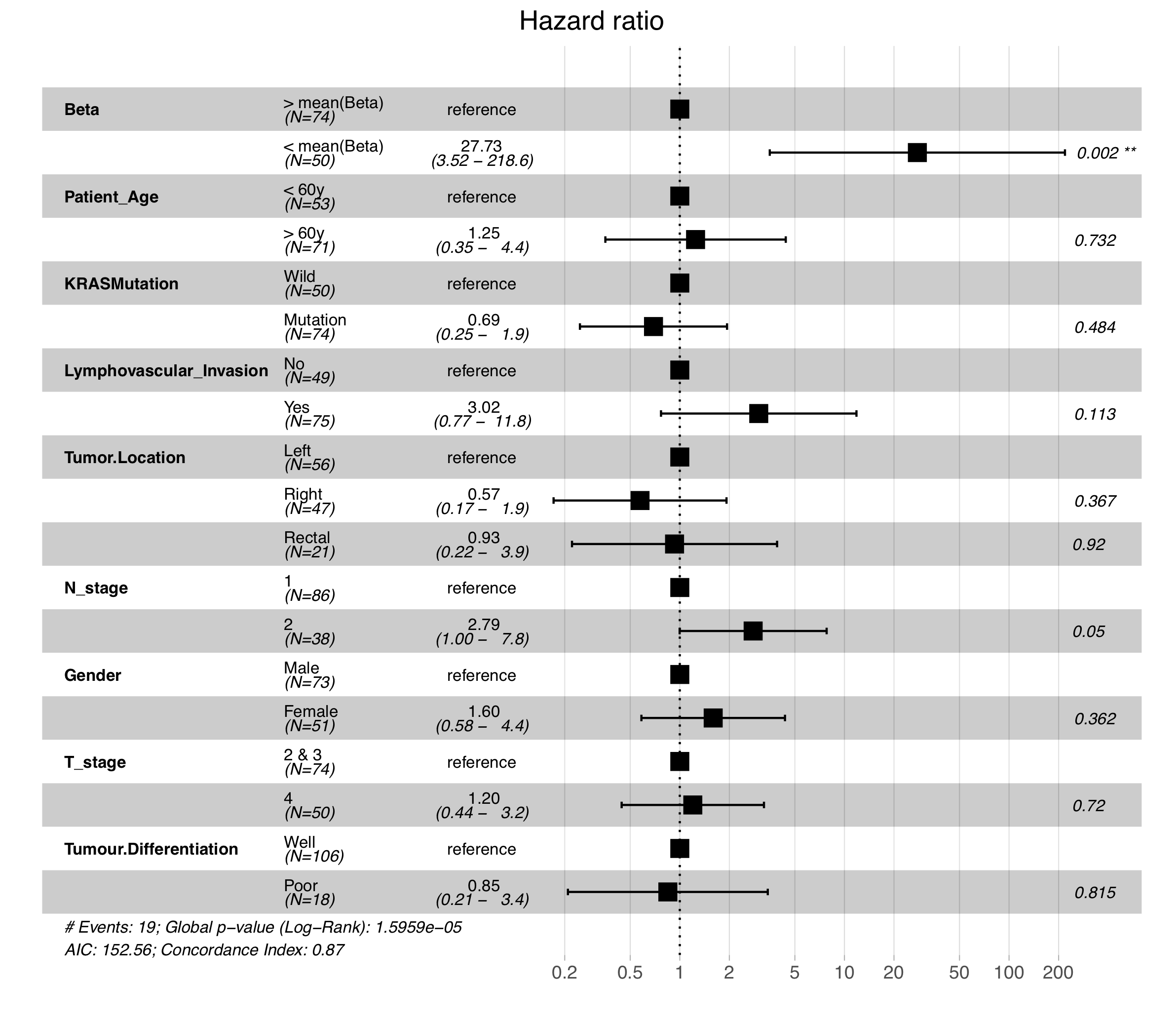

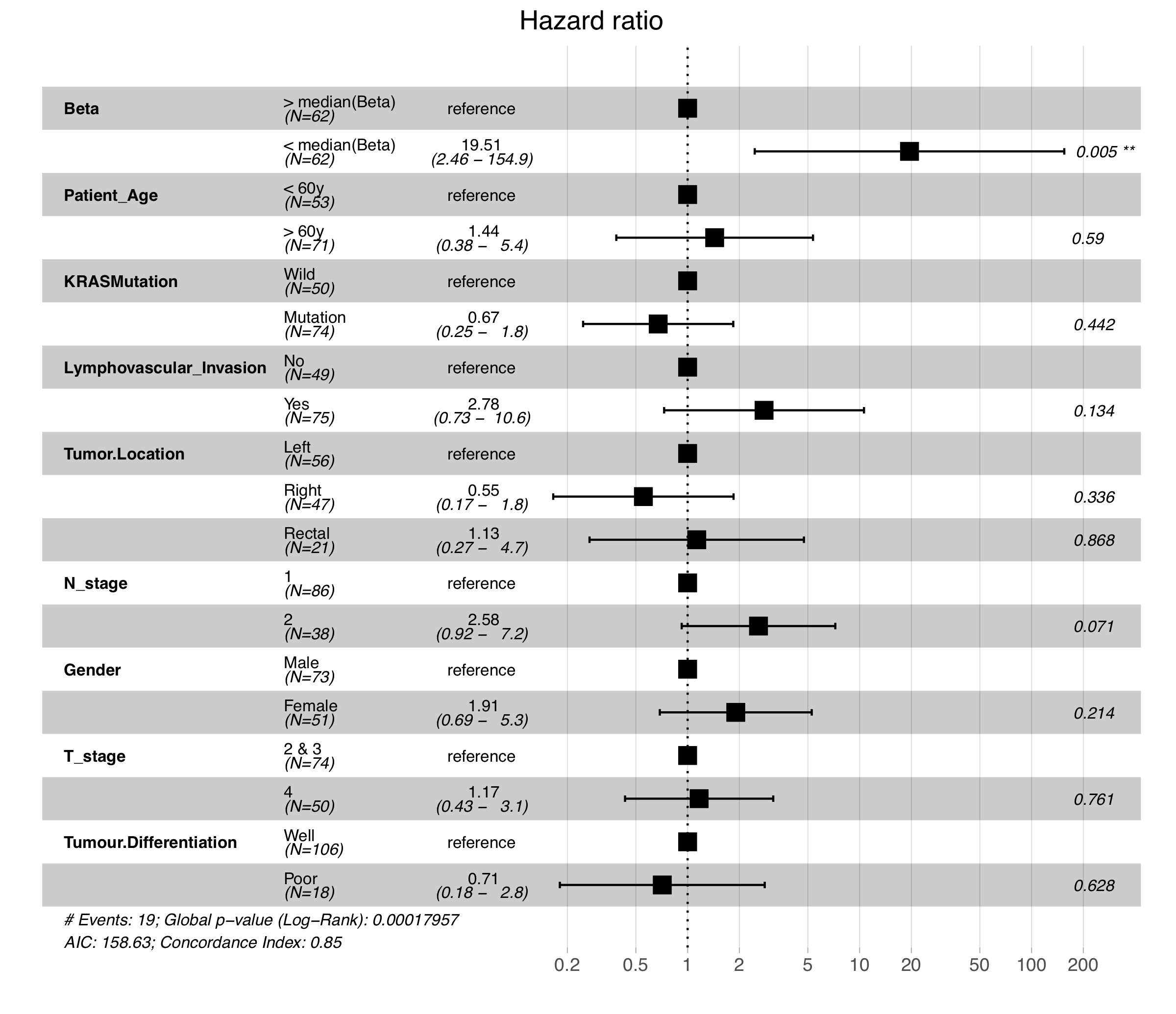


(i) (ii)

**S1 Fig G**


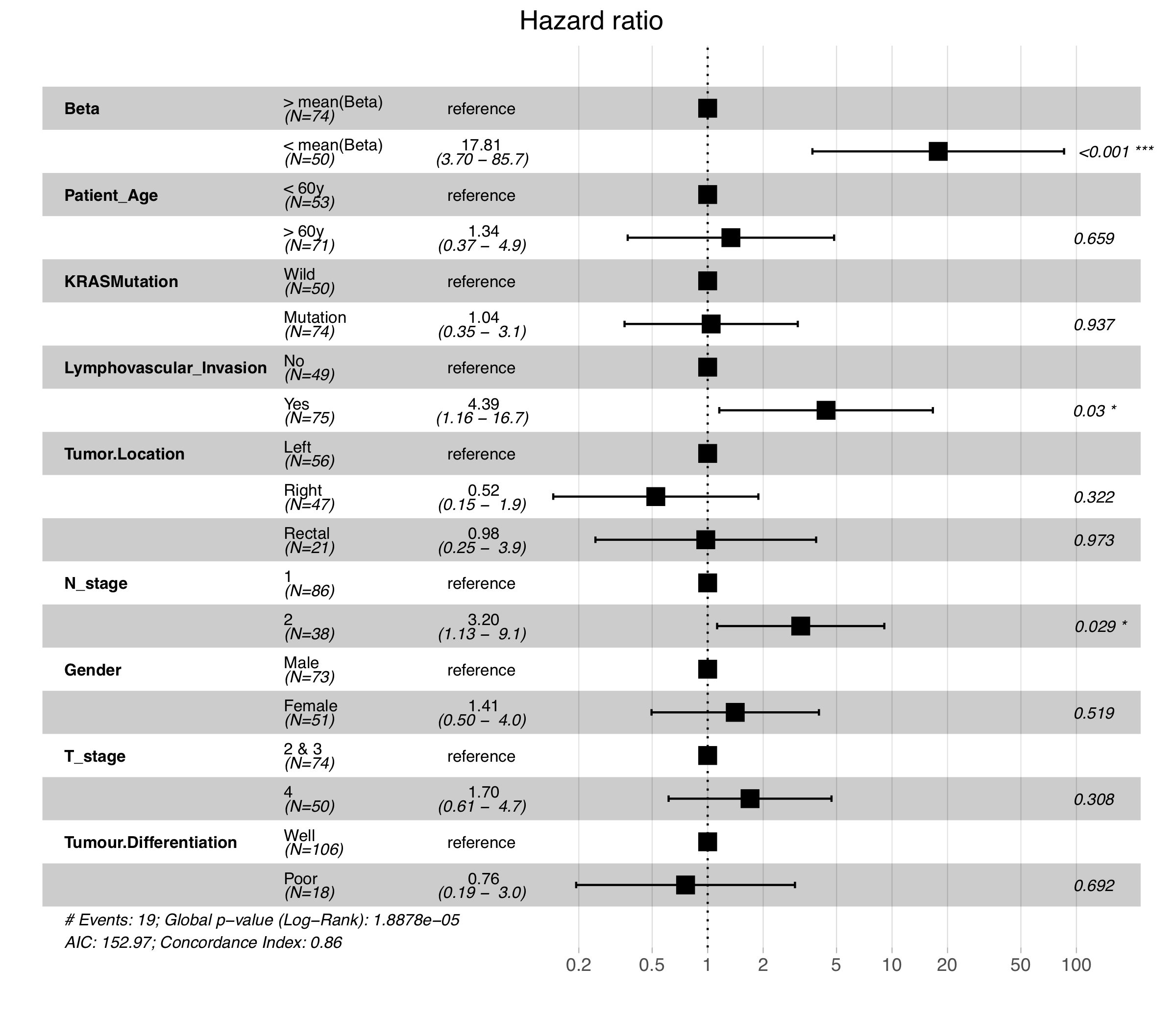

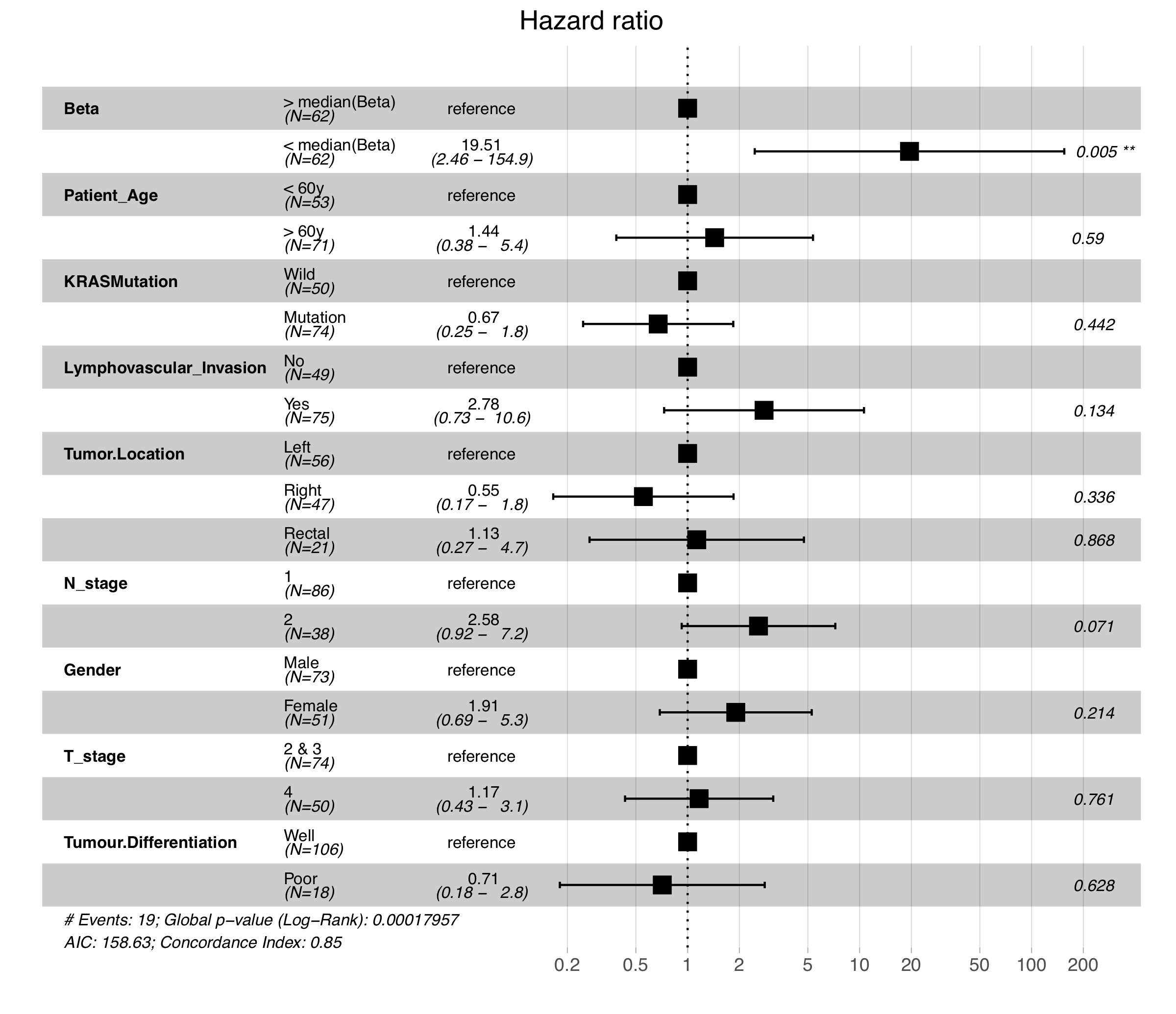


(i) (ii)

**S1 Fig H**


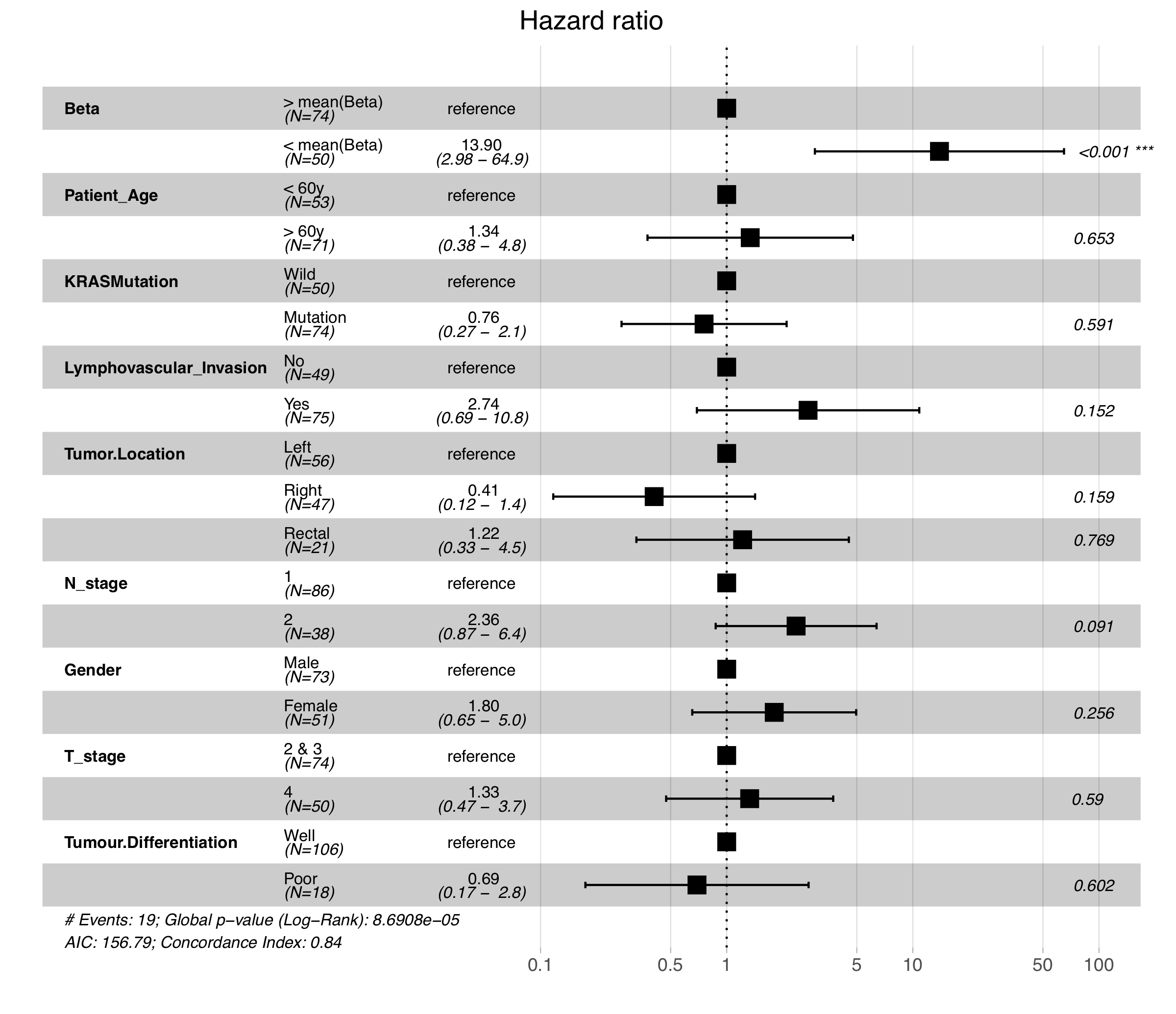

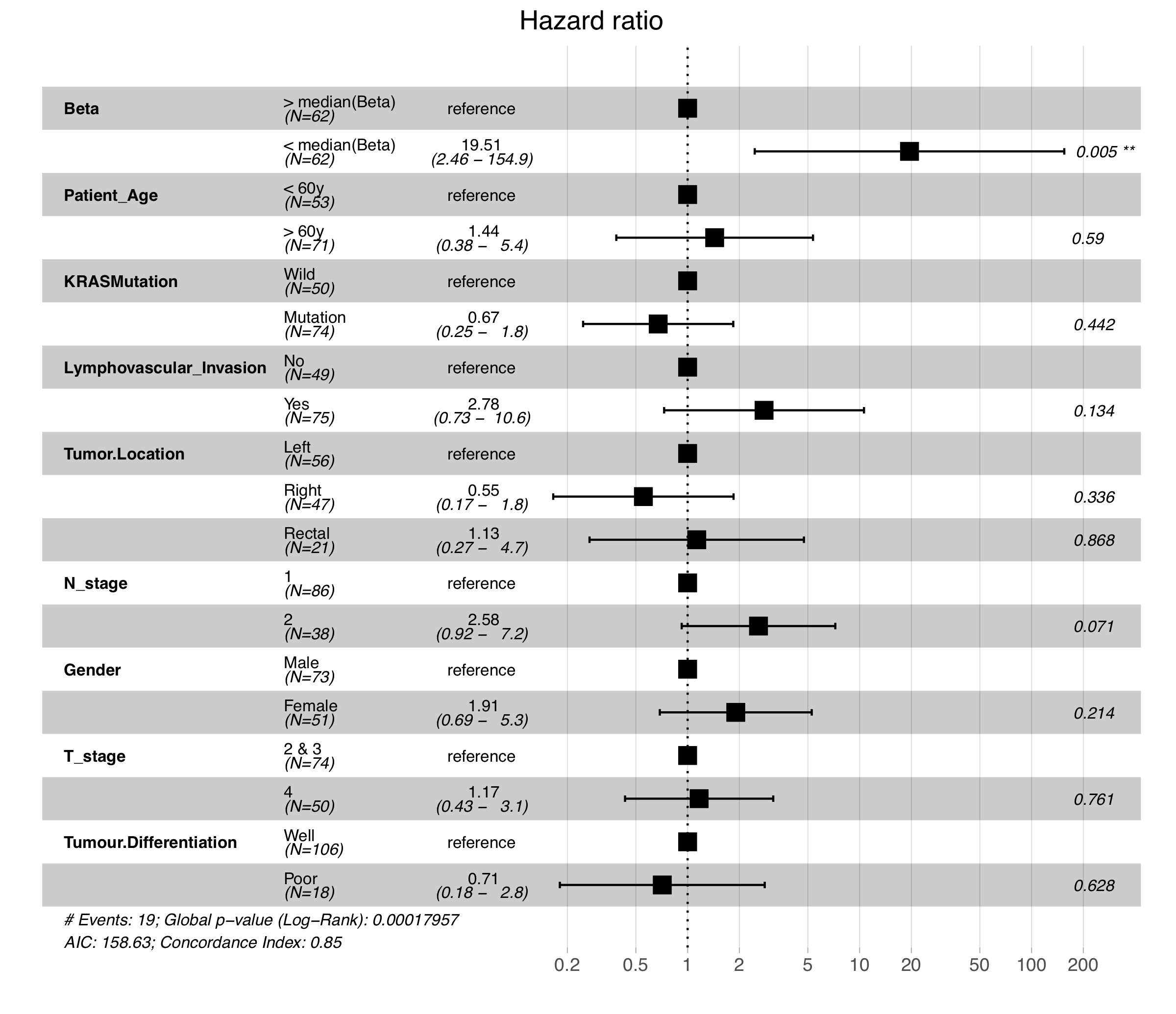


(i) (ii)

**S1 Fig I**


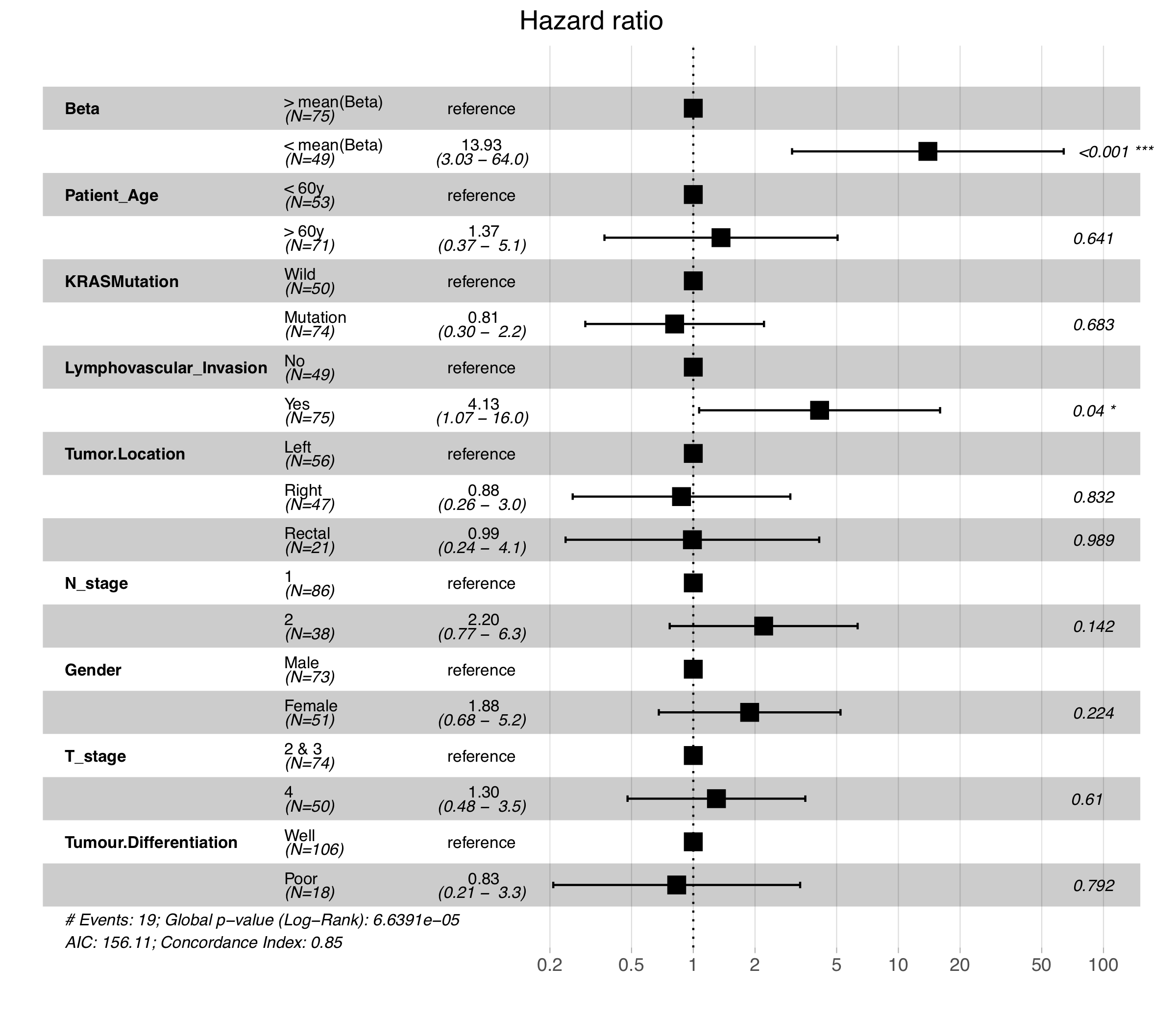

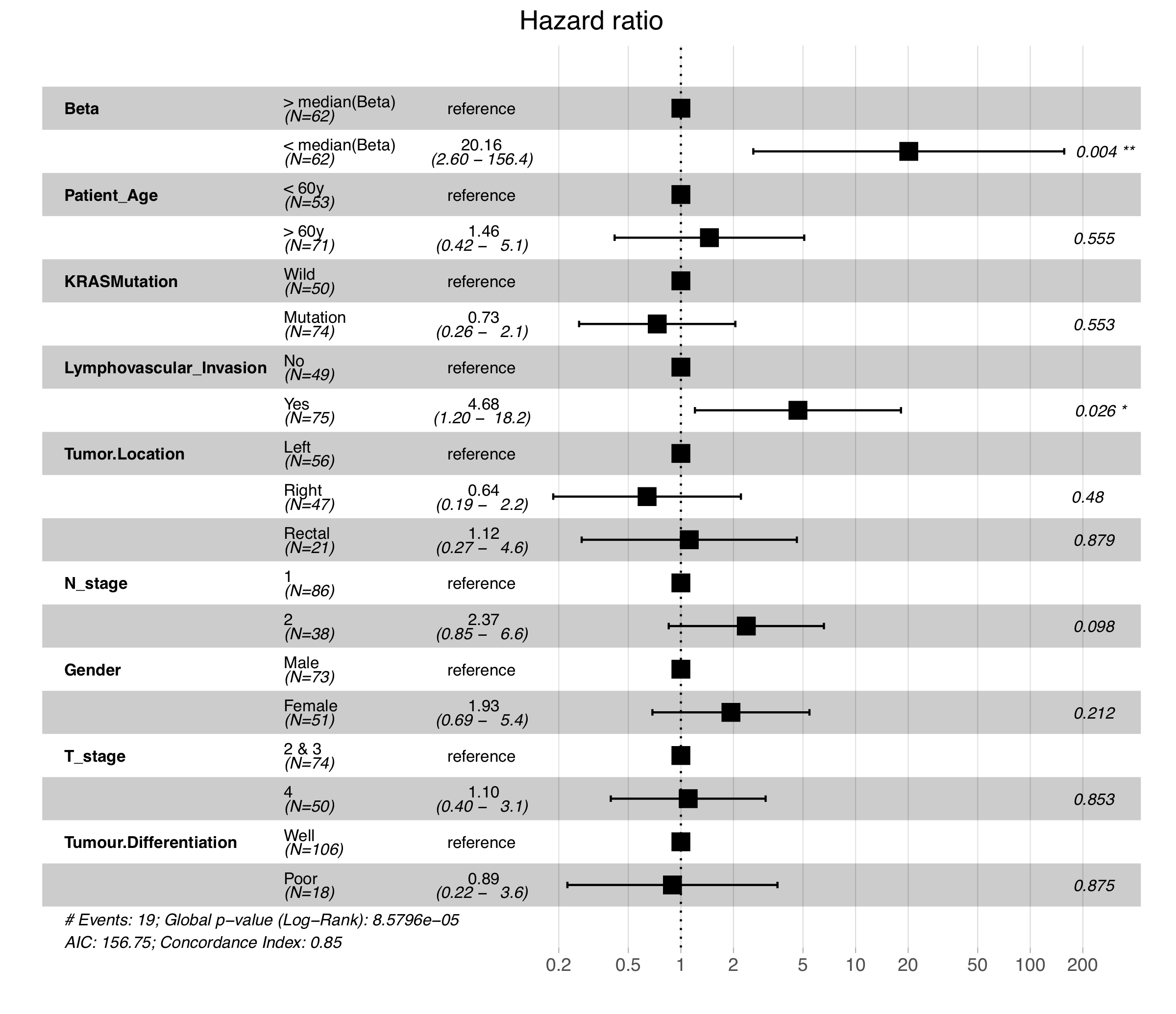


(i) (ii)

**S1 Fig J**


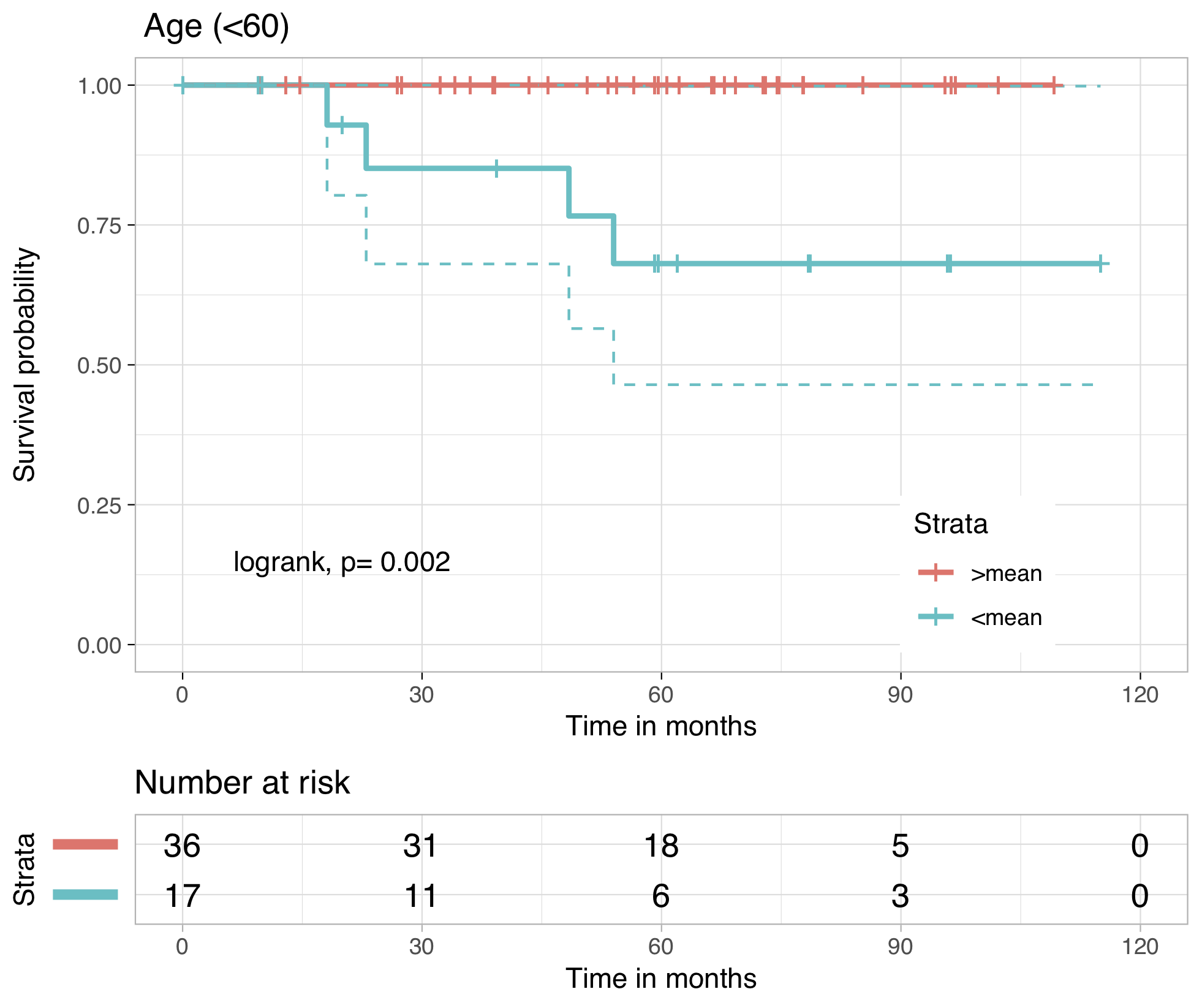

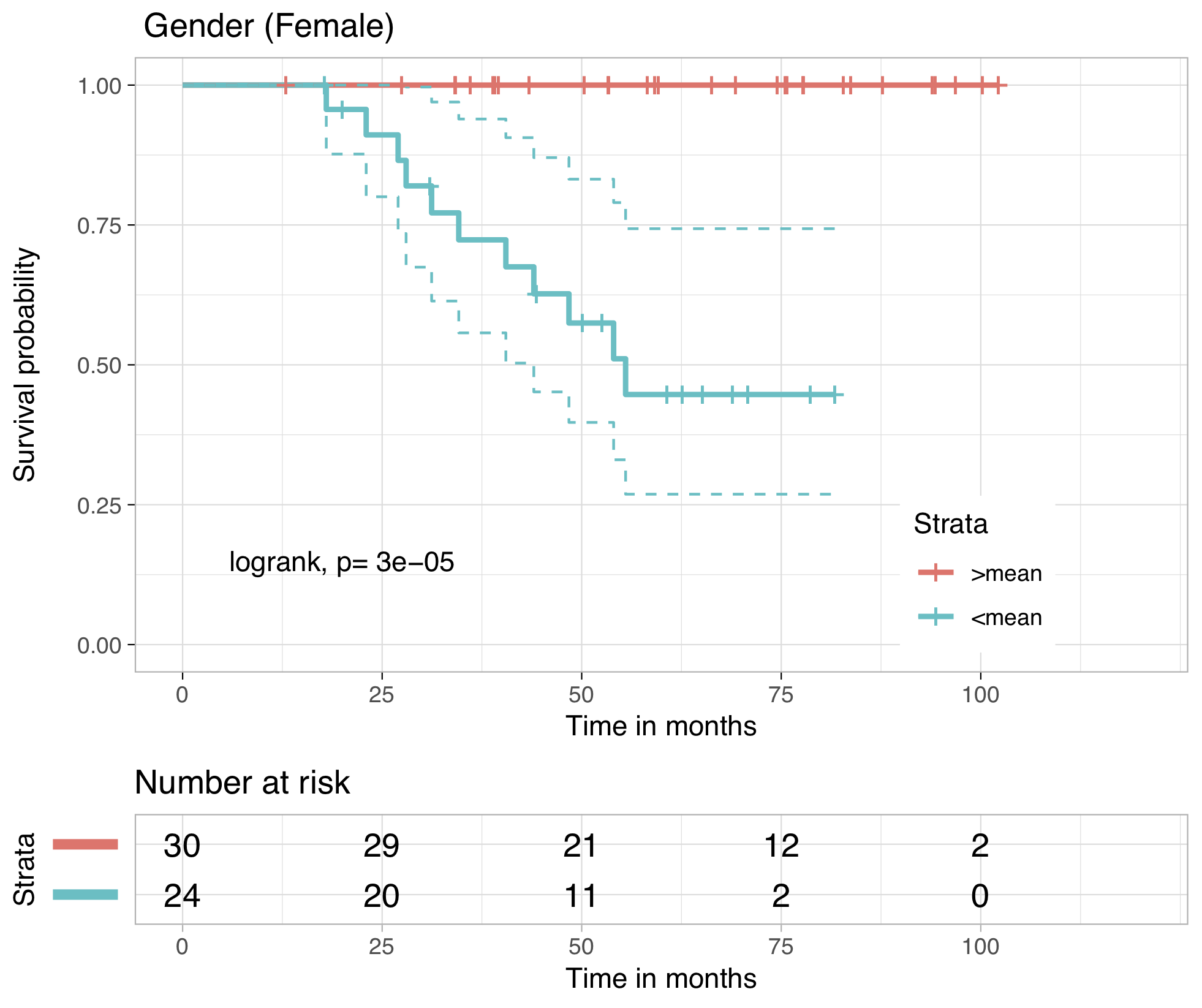


(i) (ii)


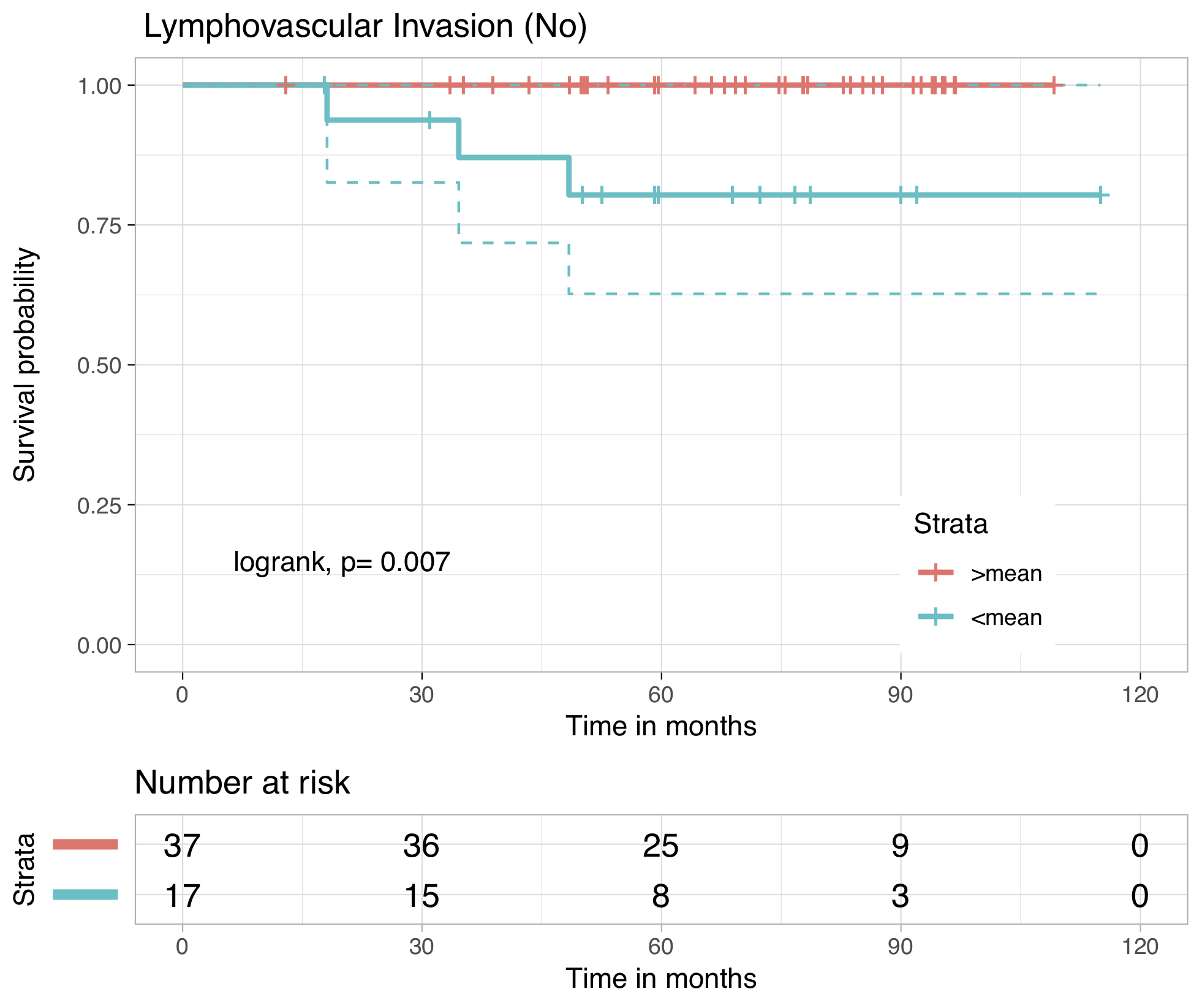

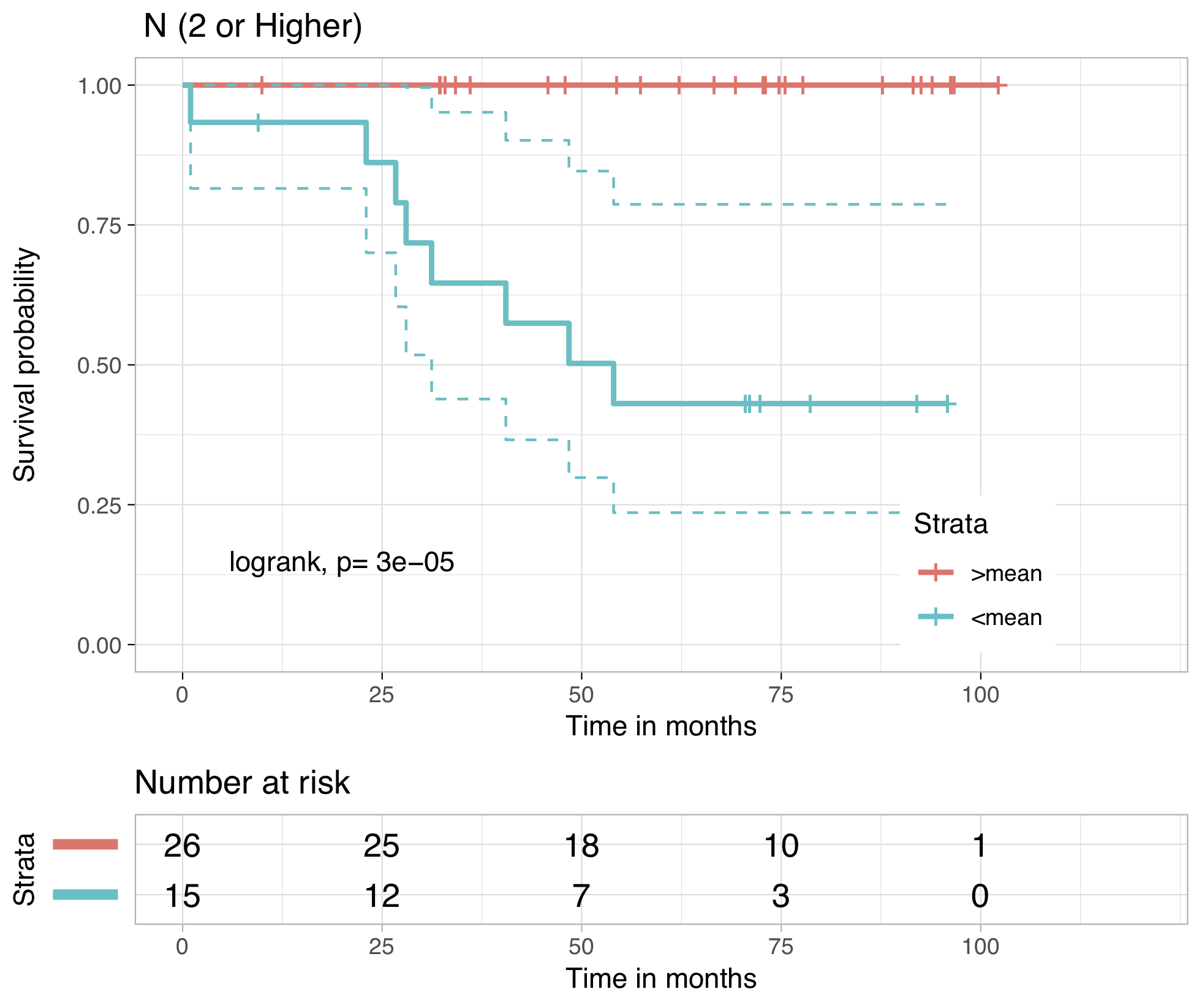


(iii) (iv)

**S1 Fig K**


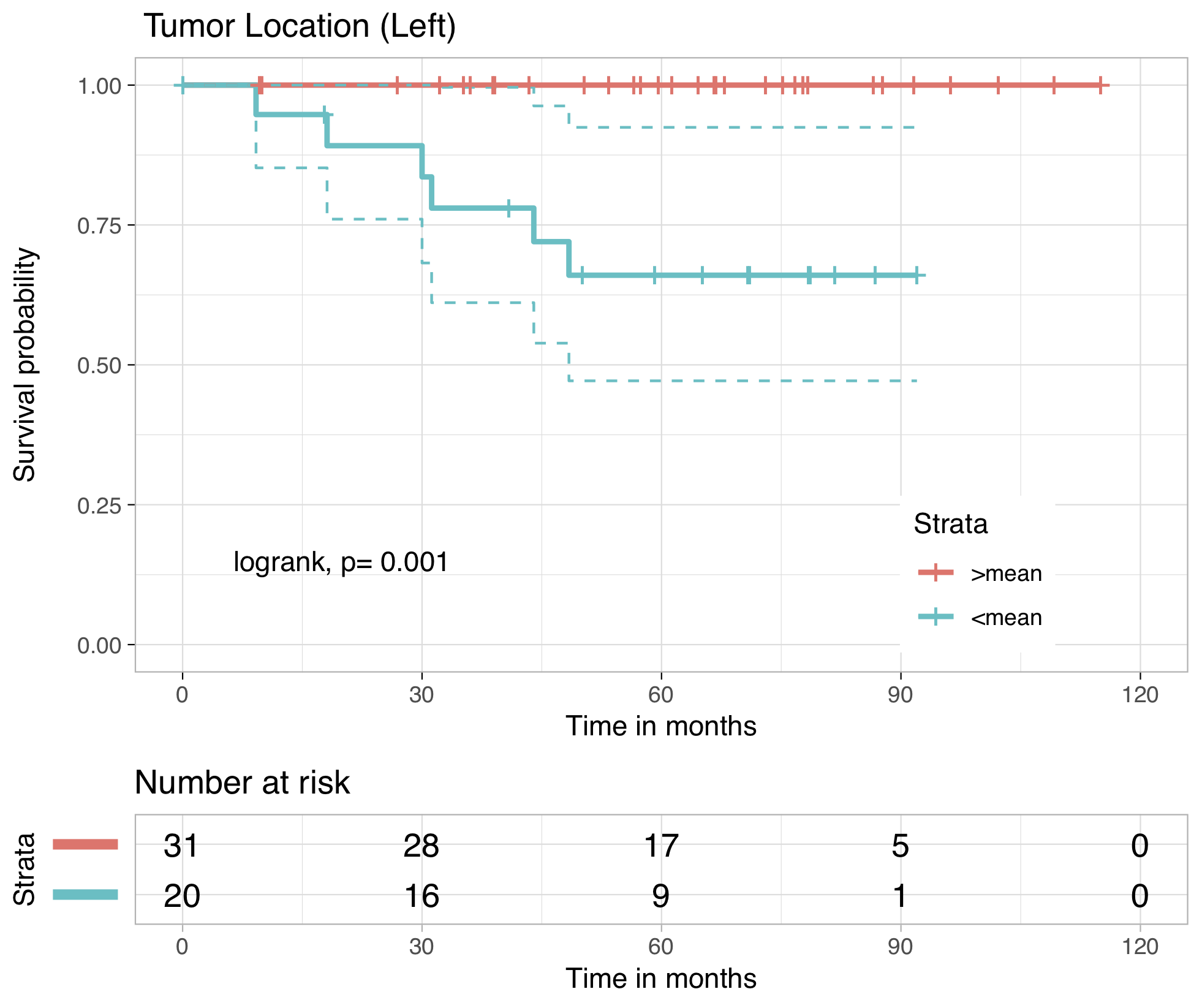

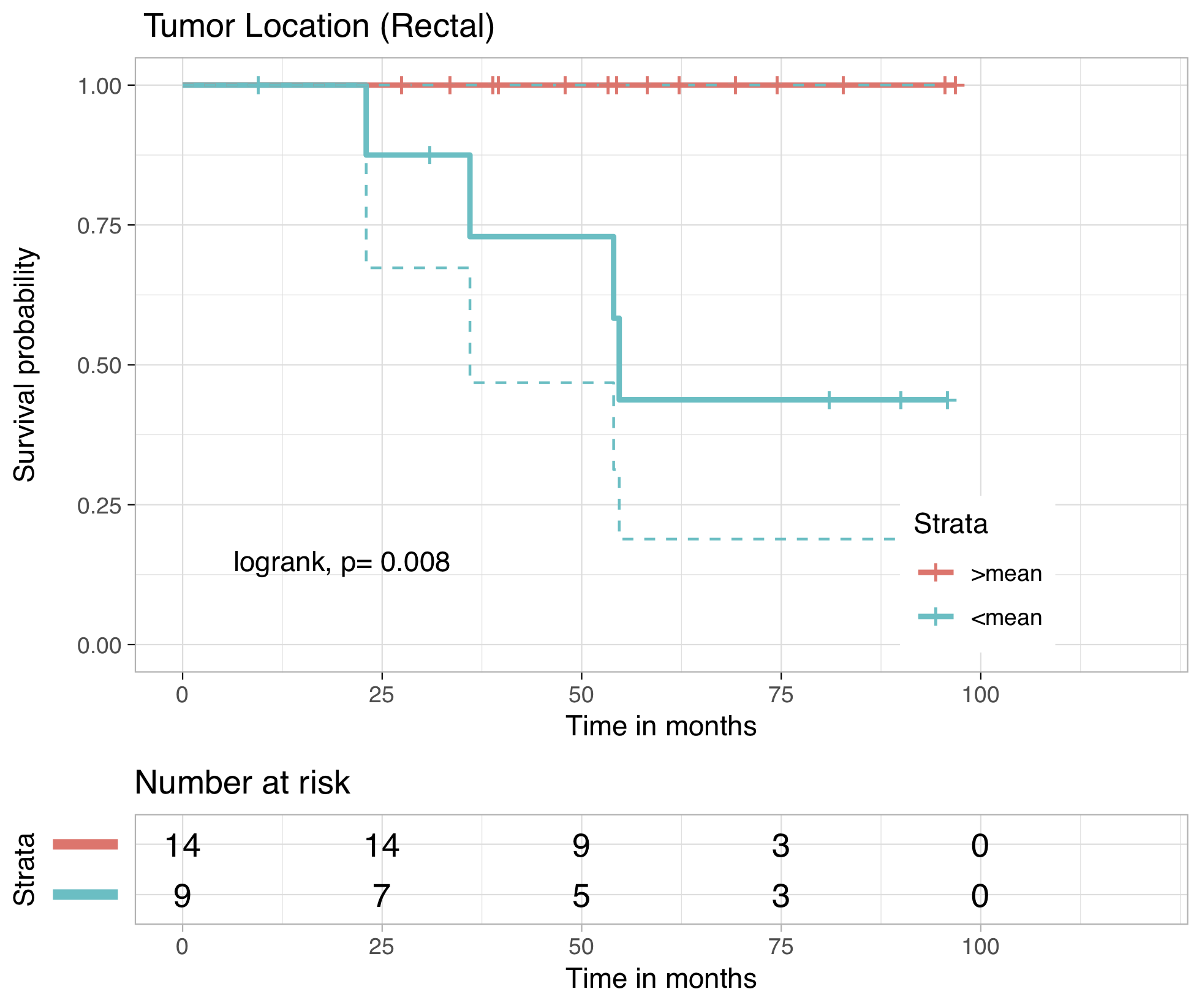


(i) (ii)


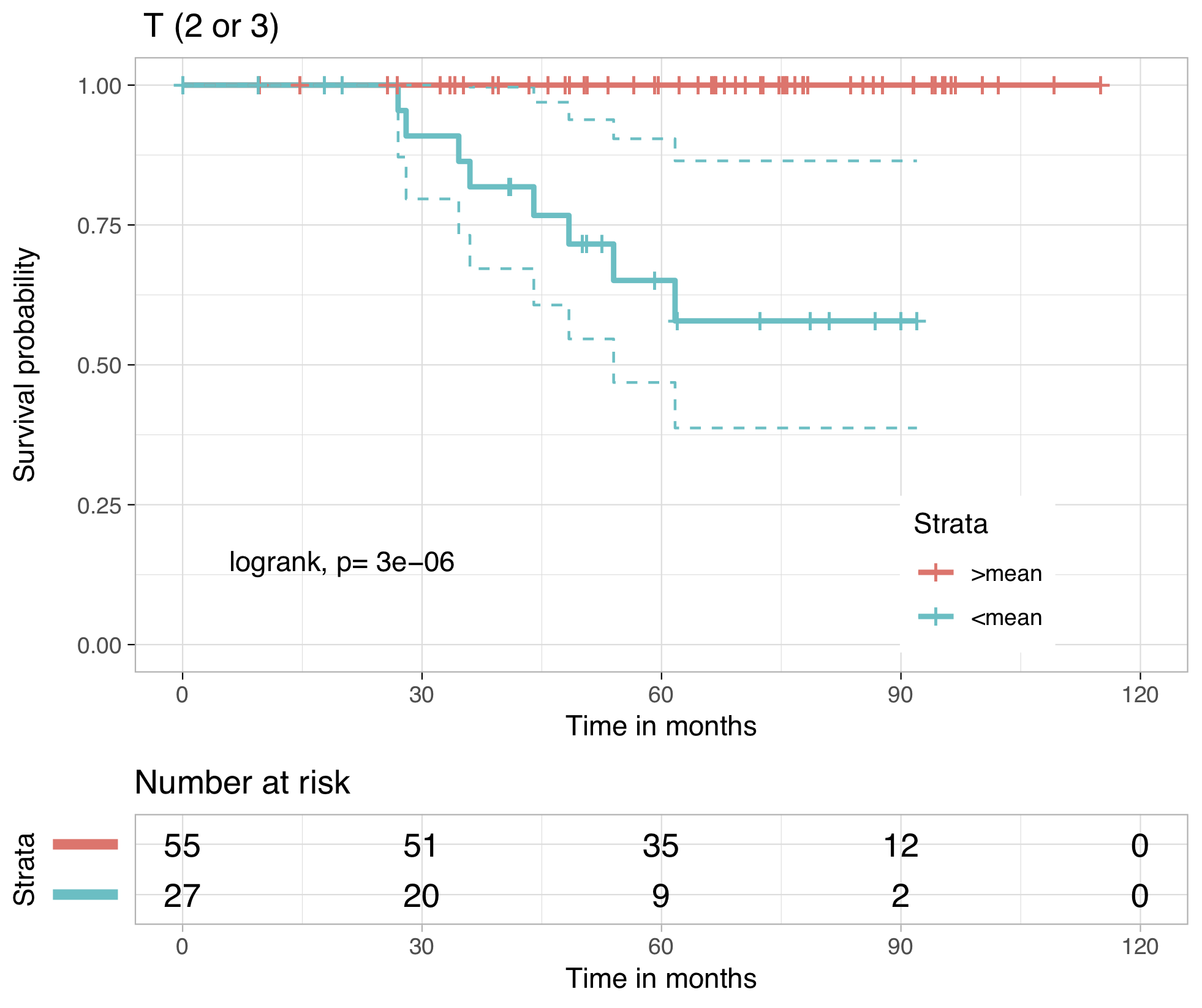


(iii)

**S1 Fig L**
